# Post-Surgical Pyoderma Gangrenosum After Breast Cancer Surgery: A Multidisciplinary Case Report

**DOI:** 10.3390/curroncol32120701

**Published:** 2025-12-12

**Authors:** Raquel Diaz, Rebecca Allievi, Letizia Cuniolo, Maria Stella Leone, Ilaria Baldelli, Federica Toscanini, Giulia Buzzatti, Andrea Bellodi, Chiara Cornacchia, Federica Murelli, Francesca Depaoli, Cecilia Margarino, Chiara Boccardo, Marco Gipponi, Marianna Pesce, Simonetta Franchelli, Amandine Causse d’Agraives, Piero Fregatti

**Affiliations:** 1 Department of Surgical Sciences and Integrated Diagnostic (DISC), University of Genoa, 16132 Genoa, Italy; 2 Breast Surgery Unit, IRCCS Ospedale Policlinico San Martino, 16132 Genoa, Italy; 3 Plastic and Reconstructive Surgery Unit, IRCCS Ospedale Policlinico San Martino, 16132 Genoa, Italy; 4 Infectious Diseases Unit, IRCCS Ospedale Policlinico San Martino, 16132 Genoa, Italy; 5 Hereditary Cancer Unit, IRCCS Ospedale Policlinico San Martino, 16132 Genoa, Italy; 6 Department of Internal Medicine and Medical Specialties (DIMI), University of Genoa, 16132 Genoa, Italy

**Keywords:** post-surgical pyoderma gangrenosum, breast cancer surgery, multidisciplinary approach

## Abstract

Post-surgical pyoderma gangrenosum is a rare neutrophilic dermatosis that may occur after surgical procedures, mimicking a wound infection. Early recognition is crucial to prevent unnecessary debridement and worsening of lesions due to pathergy. We report the case of a 67-year-old woman who underwent nipple-sparing mastectomy for invasive breast carcinoma with immediate reconstruction using a tissue expander. In the early postoperative period, she developed an extensive sterile necrotic–ulcerative inflammation of the left breast, unresponsive to broad-spectrum antibiotics and repeated surgical revisions. Histopathology revealed an aseptic neutrophilic infiltrate, confirming the diagnosis of post-surgical pyoderma gangrenosum. The patient responded favorably to high-dose corticosteroid therapy, achieving complete wound healing and definitive reconstruction with a TRAM flap. This case highlights the importance of considering post-surgical pyoderma gangrenosum in the differential diagnosis of inflammatory postoperative complications in breast oncology surgery. Prompt diagnosis and early initiation of immunosuppressive therapy within a multidisciplinary approach are key to preserving tissues and ensuring optimal functional and aesthetic outcomes.

## Simple Summary

Post-surgical pyoderma gangrenosum is an uncommon autoinflammatory disorder that can closely mimic postoperative infection, thereby complicating timely diagnostic evaluation and management. This report describes a case of rapidly progressive postoperative inflammation following breast cancer surgery with immediate reconstruction, refractory to antimicrobial therapy and consistently negative on microbiological investigation. Histopathological examination revealed a sterile neutrophilic infiltrate, with prompt clinical improvement after immunosuppressive therapy. This case underscores the importance of considering this rare entity when postoperative inflammatory findings are incongruent with an infectious etiology.

## 1. Introduction

Breast cancer is the most frequently diagnosed malignancy among women worldwide.

Among surgical options, nipple-sparing mastectomy (NSM) has emerged as an important approach for selected patients, providing effective oncologic control while optimizing aesthetic outcomes. However, its postoperative course may be complicated by rare but potentially serious events, including post-surgical pyoderma gangrenosum (PSPG), a neutrophilic dermatosis characterized by rapidly progressive ulceration that can closely mimic surgical site infection. Although uncommon, PSPG is most often reported after breast surgery. Systematic reviews indicate that breast procedures account for approximately 25–30% of postoperative cases, making the breast the most frequent anatomic site of onset. This higher incidence is thought to relate to extensive tissue manipulation, wide cutaneous involvement, and the frequent use of reconstructive techniques, which may intensify pathergy.

Prompt recognition is crucial to prevent extensive tissue loss and preserve reconstructive outcomes. Here, we describe the case of a woman who developed PSPG following NSM with immediate implant-based reconstruction for invasive breast carcinoma.

## 2. Case Presentation

On 22 January 2025, a 67-year-old woman—with no significant comorbidities, autoimmune or inflammatory disorders, immunosuppressive conditions, or therapies affecting wound healing, but with a notable family history of breast cancer—underwent a screening mammogram that identified a 30 mm area of architectural distortion in the upper quadrants of the left breast (R4c). Targeted ultrasound confirmed the abnormality and revealed no additional lesions or axillary lymphadenopathy bilaterally (E4c).

An ultrasound-guided tru-cut core biopsy was subsequently performed, diagnosing invasive carcinoma of no special type (NST), moderately differentiated (G2), strongly positive for estrogen and progesterone receptors (ER 99%, PgR 99%), with a low proliferative index (Ki-67 12–14%) and no oncoprotein C-erbB2 overexpression (HER2 0).

Following multidisciplinary tumor board evaluation and BRCA genetic testing—negative for pathogenic germline variants—surgical treatment was recommended. On 27 February 2025, the patient underwent left NSM with ipsilateral sentinel lymph node biopsy (SLNB) and immediate implant-based reconstruction using a retro-muscular tissue expander and bovine pericardial membrane.

Perioperative prophylaxis followed institutional protocols for clean breast surgery with implant placement. A first-generation cephalosporin (cefazolin) was administered prior to incision, with additional intraoperative doses provided as needed according to the duration of the procedure.

The postoperative course was initially uneventful, and the patient was discharged in good clinical condition on postoperative day 4 (3 March 2025).

Final histopathology confirmed invasive carcinoma NST, pT2/G2/N1a/Mx. Evaluation of prognostic and predictive biomarkers on the surgical specimen demonstrated complete hormone receptor positivity (ER 100%, PgR 100%), a moderately increased proliferative index (Ki-67 20–25%), and absence of oncoprotein C-erbB2 expression (HER2 0).

On 4 March 2025, the patient developed high-grade fever associated with redness, edema, and pain of the left breast. The following day, drainage fluid culture was obtained and resulted negative. Laboratory tests showed leukocytosis (16.08 × 10^9^/L) and elevated inflammatory markers—erythrocyte sedimentation rate (ESR) 77 mm/h and C-reactive protein (CRP) 214.1 mg/L—while procalcitonin (PCT) remained within the low-risk range for sepsis (0.22 µg/L).

On 6 March 2025, due to persistent fever and worsening local inflammation, she was urgently readmitted with suspected periprosthetic infection.

Repeat laboratory tests confirmed a severe inflammatory response, with increasing leukocytosis and CRP (234.6 mg/L). Blood cultures were negative.

On 7 March 2025, after infectious disease consultation, empirical antibiotic therapy with daptomycin was initiated, and the tissue expander was surgically removed.

Microbiological cultures from drainage fluid, biopsy tissue, and prosthetic material were all negative. Despite expander removal and antibiotic therapy, local deterioration continued.

On 9 March 2025, antimicrobial treatment was intensified with piperacillin–tazobactam and clindamycin to broaden Gram-negative and anaerobic coverage in view of a possible evolving necrotizing infection. Close monitoring, including delineation of inflammatory margins and repeat imaging, was advised.

Breast ultrasound revealed marked edema without fluid collections, whereas thoraco-abdominal CT showed multiple confluent gas-fluid collections in the left breast region and a fluid collection in the ipsilateral axilla, associated with diffuse skin thickening and a small left pleural effusion.

Given the unchanged clinical picture, the infectious disease team replaced the previous regimen with meropenem and caspofungin, in addition to ongoing daptomycin.

On 11 March 2025, laboratory tests showed worsening leukocytosis (19.92 × 10^9^/L), persistently elevated CRP (202 mg/L), and hypoalbuminemia. Due to suspected soft tissue fasciitis, hyperbaric oxygen therapy was scheduled (three urgent sessions at 2.8 ATA followed by two at 2.5 ATA).

On 12 March 2025, as leukocytosis further increased to 23.29 × 10^9^/L, the patient was reassessed by infectious disease, oncology, and immunology specialists. Daptomycin was replaced with ceftaroline to address a possible resistant Gram-positive etiology. The immunologist, after excluding major lymphocytic or humoral deficiencies, suggested that the process might reflect a foreign-body–driven inflammatory reaction potentially amplified by tumor-related immune dysregulation.

**Figure 1:**
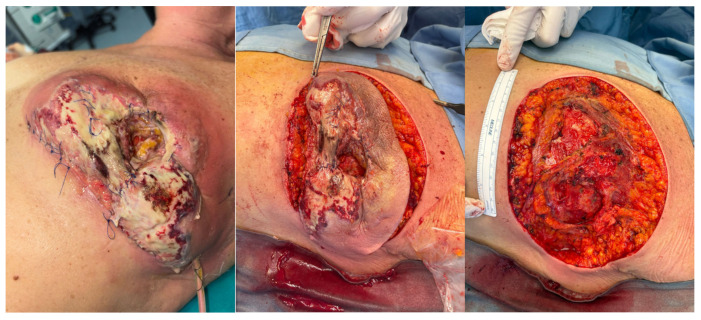
Surgical revision of the wound performed on 13 March 2025.

On 13 March 2025, surgical wound revision with placement of negative pressure wound therapy (NPWT) was performed [Figure 1]. Intraoperatively, extensive necrotic areas involving both the cutaneous and subcutaneous tissues, as well as the pectoralis major muscle, were observed, with no evidence of abscess formation. All cultures once again yielded negative results. Histopathological examination of the intraoperative samples revealed necrotizing and abscess-forming lymphogranulocytic inflammation, associated with a foreign body-type giant cell reaction, without evidence of neoplasia or microorganisms.

In the following days, progressive clinical and laboratory improvement was observed. On 15 March 2025, the white blood cell count was 7.45 × 10^9^/L and CRP 80.7 mg/L; by 17 March, values had decreased to 6.64 × 10^9^/L and 23 mg/L, respectively, and beta-D-glucan tested negative.

On 19 March, the first NPWT replacement showed a well-granulating wound bed. Given the near-normalization of inflammatory markers, meropenem and caspofungin were discontinued, with continuation of ceftaroline alone.

On 25 March, a second NPWT replacement with wound curettage and flap approximation at the muscular planes was performed.

The patient was discharged the following day in good clinical condition. Despite multidisciplinary evaluation by infectious disease, oncology, and immunology specialists, no infectious or systemic cause was identified to explain the necrotic–inflammatory process.

**Figure 2:**
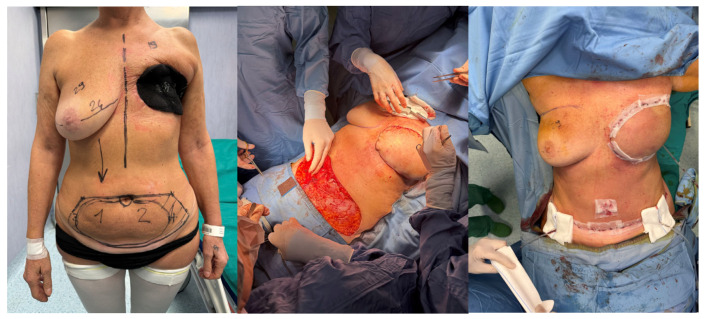
Autologous breast reconstruction with a TRAM flap performed on 10 April 2025.

After a short hospital stay for a third NPWT system replacement—completed without complications—the patient underwent routine preoperative testing on 9 April 2025, all within normal limits, in preparation for the planned reconstructive surgery. On 10 April 2025, she underwent left autologous breast reconstruction with a TRAM flap [Figure 2].

On the first postoperative day, she developed a left corneal abrasion, treated with topical therapy, along with intermittent fever and erythema over the abdominal flap.

On 13 April 2025, blood cultures were obtained and resulted negative.

The following day, she experienced another febrile episode with leukocytosis (10.43 × 10^9^/L) and elevated CRP (185.2 mg/L). Both blood cultures and beta-D-glucan testing were negative. Given the complexity of the clinical picture, the infectious disease specialist recommended initiating ceftaroline.

On 15 April, contrast-enhanced CT scans of the brain and thoraco-abdominal regions were performed. Cranial CT was unremarkable, whereas thoraco-abdominal CT showed persistent confluent blood and gas–fluid collections in the left breast and abdominal wall, without organized abscesses. Additional findings included a small left pleural effusion and thickening of the right rectus abdominis muscle. On the same day, an internal medicine specialist evaluated the patient and considered the findings consistent with a postoperative aseptic autoinflammatory reaction suggestive of pyoderma gangrenosum. Given the risk of rapid progression, systemic corticosteroid therapy at an immunosuppressive intravenous dose (1 mg/kg/day) was initiated.

On 16 April 2025, with early clinical improvement, the infectious disease specialist advised discontinuing ceftaroline. The internist proposed that the process had been triggered by a pathergic reaction following the core biopsy and subsequently amplified by surgical stress, recommending continuation of corticosteroid therapy for an additional five days with monitoring of inflammatory markers and renal function.

In the following days, the patient showed steady clinical and laboratory improvement.

On 22 April 2025, she underwent surgical revision of the TRAM flap with placement of a new NPWT system and remained afebrile throughout the postoperative course.

Given the continued normalization of laboratory values (CRP 7.8 mg/L), the following day the infectious disease specialist recommended transitioning to oral corticosteroids with a gradual taper and planned biochemical follow-up.

The patient was subsequently discharged in good clinical condition.

On 28 April 2025, the patient underwent the first plastic surgery wound dressing, which confirmed good local and overall clinical conditions. Repeat laboratory tests performed the same day were essentially normal, with a CRP level of 3.2 mg/L.

## 3. Discussion

PSPG is a rare but severe inflammatory complication that may occur after surgical procedures. It is characterized by a dysregulated innate immune response and a sterile neutrophilic infiltrate, leading to rapidly progressive ulceration of the skin and subcutaneous tissues. Although described in different surgical settings, breast surgery accounts for up to 25% of reported postoperative cases [1,2,3]. Early recognition is crucial, as PSPG closely mimics surgical site infection and often prompts repeated debridement or antibiotic escalation—interventions that may exacerbate the condition through pathergy [4,5].

In the present case, five days after left NSM with SLNB and immediate prosthetic reconstruction, the patient developed severe inflammation with extensive necrosis, fever, leukocytosis, and markedly elevated CRP, despite consistently negative cultures.

The extension progression of the lesions despite broad-spectrum antibiotic therapy antibiotics and repeated surgical debridement revisions, together with persistently negative culture sterility and the subsequent favorable response to immunomodulatory therapy, supported the diagnosis of PSPG.

This timeline mirrors what is reported in the literature, where onset typically occurs between 4 and 10 days postoperatively, and diagnosis is frequently delayed beyond the first week due to ineffective antibiotic treatment [6,7].

Pathophysiologically, pyoderma gangrenosum is an autoinflammatory neutrophilic dermatosis marked by innate immune dysregulation, neutrophil hyperactivation, and overexpression of cytokines such as TNF-α, IL-1β, IL-17, and IL-23 [8,9]. In postoperative cases, surgical trauma can trigger pathergy, inducing an exaggerated inflammatory response even in the absence of infection [10]. In our patient—who had no systemic predisposing conditions—the aseptic inflammatory reaction following repeated procedures is consistent with a pathergy-driven mechanism: the core needle biopsy likely acted as an initial priming event, while NSM and expander placement amplified the inflammatory cascade, resulting in extensive tissue destruction in the absence of pathogens.

The diagnosis of PSPG is challenging and remains largely one of exclusion.

The criteria proposed by Su et al. (2004) and the 2018 Delphi Consensus—featuring one major histopathologic criterion (biopsy demonstrating a sterile neutrophilic infiltrate) and several supportive clinical features—provide a structured diagnostic approach [7,11].

The PARACELSUS score, introduced in 2019, further aids differentiation from necrotizing infections [12,13]. In our case, histopathology confirmed a sterile neutrophilic infiltrate with a foreign body-type giant cell reaction, fulfilling the major criterion. Repeatedly negative cultures, post-traumatic evolution, and rapid steroid response reinforced the diagnosis.

Distinguishing PSPG from necrotizing infections is critical but difficult. In such infections, the course is typically dominated by systemic toxemia, hypotension, and radiologic findings of extensive fascial gas, whereas in PSPG, the absence of sepsis, low procalcitonin levels, and lack of identifiable pathogens point toward an autoinflammatory process [14]. Imaging may show fluid or gas collections, but these findings are nonspecific and must be interpreted clinically [15]. In our patient, the discordance between the severity of the presentation and the lack of microbiologic evidence helped redirect the workup toward a non-infectious diagnosis.

Management of PSPG relies on early systemic immunosuppression to halt neutrophil-driven inflammation and prevent further tissue loss. Corticosteroids are the first-line therapy; the STOP GAP trial demonstrated comparable efficacy between prednisolone (0.75 mg/kg/day) and cyclosporine (4 mg/kg/day) [16,17]. In this case, high-dose intravenous methylprednisolone led to rapid improvement.

For recurrent forms, biologic agents targeting TNF-α (infliximab, adalimumab), IL-1β (anakinra, canakinumab), or IL-23 (ustekinumab) have shown response rates exceeding 70% in observational studies [18,19]; intravenous immunoglobulins (IVIG) may also be considered in selected patients [20,21].

From a surgical standpoint, extensive debridement should be avoided during the active inflammatory phase due to the high risk of pathergy. A conservative strategy, involving selective tissue removal, NPWT, and adequate immunosuppression, is associated with more favorable outcomes [22,23]. Definitive reconstruction is best deferred until full disease quiescence, preferably under corticosteroid coverage.

In our patient, the combined strategy of systemic immunosuppression, surgical revision of the TRAM flap, and NPWT placement resulted in complete clinical resolution and satisfactory reconstructive results, underscoring the value of a multidisciplinary approach.

## 4. Conclusions

This case underscores the need to consider PSPG in the differential diagnosis of postoperative infections, particularly after breast surgery.

Early recognition, a coordinated multidisciplinary approach involving surgical and medical specialists, and timely initiation of immunosuppressive therapy are essential to avoid unnecessary procedures and improve outcomes. Furthermore, the clinical course demonstrates that breast reconstruction can be successfully achieved even in the setting of PSPG, provided the condition is promptly identified and effectively managed.

## Data Availability

The data presented in this study are available in this article.

## References

[1] Zuo K.J., Fung E., Tredget E.E., Lin A.N. (2015). A systematic review of post-surgical pyoderma gangrenosum: Identification of risk factors and proposed management strategy. J. Plast. Reconstr. Aesthet. Surg..

[2] Ehrl D.C., Heidekrueger P.I., Broer P.N. (2018). Pyoderma gangrenosum after breast surgery: A systematic review. J. Plast. Reconstr. Aesthet. Sur..

[3] Tuffaha S.H., Sarhane K.A., Mundinger G.S., Broyles J.M., Reddy S.K., Azoury S.C., Seal S., Cooney D.S., Bonawitz S.C. (2016). Pyoderma gangrenosum after breast surgery: Diagnostic pearls and treatment recommendations based on a systematic literature review. Ann. Plast. Surg..

[4] Edinger K.M., Rao V.K. (2022). The Management of postsurgical pyoderma gangrenosum following breast surgery. Plast. Reconstr. Surg. Glob. Open.

[5] Tolkachjov S.N., Fahy A.S., Wetter D.A., Brough K.R., Bridges A.G., Davis M.D., El-Azhary R.A., McEvoy M.T., Camilleri M.J. (2015). Postoperative pyoderma gangrenosum (PG): The Mayo Clinic experience of 20 years from 1994 through 2014. J. Am. Acad. Dermatol..

[6] Binus A.M., Qureshi A.A., Li V.W., Winterfield L.S. (2011). Pyoderma gangrenosum: A retrospective review of patient characteristics, comorbidities and therapy in 103 patients. Br. J. Dermatol..

[7] Su W.P., Davis M.D., Weenig R.H., Powell F.C., Perry H.O. (2004). Pyoderma gangrenosum: Clinicopathologic correlation and proposed diagnostic criteria. Int. J. Dermatol..

[8] Ahronowitz I., Harp J., Shinkai K. (2012). Etiology and management of pyoderma gangrenosum: A comprehensive review. Am. J. Clin. Dermatol..

[9] Park A.N., Raj A., Bajda J., Gorantla V.R. (2024). Narrative review: Pyoderma gangrenosum. Cureus.

[10] George C., Deroide F., Rustin M. (2019). Pyoderma gangrenosum—A guide to diagnosis and management. Clin. Med..

[11] Maverakis E., Ma C., Shinkai K., Fiorentino D., Callen J.P., Wollina U., Marzano A.V., Wallach D., Kim K., Schadt C. (2018). Diagnostic criteria of ulcerative pyoderma gangrenosum: A Delphi Consensus of international experts. JAMA Dermatol..

[12] Jockenhöfer F., Wollina U., Salva K.A., Benson S., Dissemond J. (2019). The PARACELSUS score: A novel diagnostic tool for pyoderma gangrenosum. Br. J. Dermatol..

[13] Haag C., Hansen T., Hajar T., Latour E., Keller J., Shinkai K., Ortega-Loayza A.G. (2021). Comparison of three diagnostic frameworks for pyoderma gangrenosum. J. Investig. Dermatol..

[14] Bisarya K., Azzopardi S., Lye G., Drew P.J. (2011). Necrotizing fasciitis versus pyoderma gangrenosum: Securing the correct diagnosis! A case report and literature review. Eplasty.

[15] Demirdover C., Geyik A., Vayvada H. (2019). Necrotising fasciitis or pyoderma gangrenosum: A fatal dilemma. Int. Wound J..

[16] Ormerod A.D., Thomas K.S., Craig F.E., Mitchell E., Greenlaw N., Norrie J., Mason J.M., Walton S., Johnston G.A., Williams H.C. (2015). UK Dermatology Clinical Trials Network’s STOP GAP Team. Comparison of the two most commonly used treatments for pyoderma gangrenosum: Results of the STOP GAP randomised controlled trial. BMJ.

[17] Mason J.M., Thomas K.S., Ormerod A.D., Craig F.E., Mitchell E., Norrie J., Williams H.C. (2017). UK Dermatology Clinical Trials Network’s STOP GAP team. Ciclosporin compared with prednisolone therapy for patients with pyoderma gangrenosum: Cost-effectiveness analysis of the STOP GAP trial. Br. J. Dermatol..

[18] Brooklyn T.N., Dunnill M.G., Shetty A., Bowden J.J., Williams J.D., Griffiths C.E., Forbes A., Greenwood R., Probert C.S. (2006). Infliximab for the treatment of pyoderma gangrenosum: A randomised, double blind, placebo controlled trial. Gut.

[19] Maronese C.A., Pimentel M.A., Li M.M., Genovese G., Ortega-Loayza A.G., Marzano A.V. (2022). Pyoderma Gangrenosum: An updated literature Review on established and emerging pharmacological treatments. Am. J. Clin. Dermatol..

[20] Song H., Lahood N., Mostaghimi A. (2018). Intravenous immunoglobulin as adjunct therapy for refractory pyoderma gangrenosum: Systematic review of cases and case series. Br. J. Dermatol..

[21] Ronicke M., Sollfrank L., Vitus M.V., Walter L.J., Krieter M., Moelleken M., Dissemond J., Schultz E., Lauffer F., von den Driesch P. (2025). Intravenous Immunoglobulin Therapy for pyoderma gangrenosum: A multicenter retrospective analysis in 81 patients. Am. J. Clin. Dermatol..

[22] Eisendle K., Thuile T., Deluca J., Pichler M. (2020). Surgical treatment of pyoderma gangrenosum with negative pressure wound therapy and skin grafting, including xenografts: Personal experience and comprehensive review on 161 Cases. Adv. Wound Care.

[23] Almeida I.R., Coltro P.S., Gonçalves H.O.C., Westin A.T., Almeida J.B., Lima R.V.K.S., Silva M.F., Farina Junior J.A. (2021). The role of negative pressure wound therapy (NPWT) on the treatment of pyoderma gangrenosum: A systematic review and personal experience. Wound Repair. Regen..

